# FILLING GAPS FOR OLDER OHIOANS WITH LOCALLY RAISED FUNDS

**DOI:** 10.1093/geroni/igae098.2209

**Published:** 2024-12-31

**Authors:** Bailee Brekke, Leah Janssen, Oksana Dikhtyar, Matt Nelson, Robert Applebaum

**Affiliations:** Miami University, W. College Corner, Indiana, United States; Miami University, Oxford, Ohio, United States; Miami University, Oxford, Ohio, United States; Miami University, Oxford, Ohio, United States; Miami University, Oxford, Ohio, United States

## Abstract

Most older Americans aging in place at home do not financially qualify for support through the Medicaid program, and funding provided through the Older Americans Act is insufficient. As a result, 15 states have turned to local revenue sources to fill service gaps for individuals living in the community, who require social care to support independence. Ohio has a long history of utilizing such funds, generating over $200 million, more than the other 14 states combined. Despite the demonstrated success of these programs, local levy programs in Ohio do experience an array of implementation challenges. This study conducted 15 qualitative descriptive interviews with 20 program officials from various locally funded levy programs across Ohio. Thematic analysis revealed barriers, including increasing state and federal eligibility requirements, staffing issues, rural access to resources, and challenges in setting reimbursement rates. These barriers are coupled with added pressure from county officials to move individuals onto state funded programs (e.g., Medicaid HCBS Waiver programs), rather than keeping them on locally funded programs. The aim of this paper is to examine challenges that local programs are facing and provide program and policy recommendations. Findings reveal the important role local levy programs play in addressing gaps in service and the importance of local advocacy and education to continue passing these levies. Increasing communication between levy staff across the state, integration of cross-funding streams allocated by the state and increasing reimbursement rates are needed to ensure the success of the local levy programs for the future.

